# Prevalence and factors associated with chronic kidney disease among medical inpatients at the Kenyatta National Hospital, Kenya, 2018: a cross-sectional study

**DOI:** 10.11604/pamj.2019.33.321.18114

**Published:** 2019-08-23

**Authors:** Valerian Mwenda, Jane Githuku, Gladwell Gathecha, Benjamin Maranga Wambugu, Zeinab Gura Roka, Willis Owino Ong'or

**Affiliations:** 1 Field Epidemiology and Laboratory Training Programme, Ministry of Health, Nairobi, Kenya; 2 Division of Non-communicable Diseases, Ministry of Health, Nairobi Kenya; 3 Department of Clinical Medicine, Kenyatta National Hospital, Nairobi, Kenya; 4 College of Health Sciences, Moi University, Eldoret, Kenya

**Keywords:** Chronic kidney disease, inpatients, referral hospital, stage, Kenya

## Abstract

**Introduction:**

The burden of chronic kidney disease (CKD) is increasing worldwide. Few studies in low and low-middle income countries have estimated the prevalence of CKD. We aimed to estimate prevalence and factors associated with CKD among medical inpatients at the largest referral hospital in Kenya.

**Methods:**

We conducted a cross-sectional study among medical inpatients at the Kenyatta National Hospital. We used systematic sampling and collected demographic information, behavioural risk factors, medical history, underlying conditions, laboratory and imaging workup using a structured questionnaire. We estimated glomerular filtration rate (GFR) in ml/min/1.73m^2^ classified into 5 stages; G1 (≥ 90), G2 (60-89), G3a (45-59), G3b (30-44), G4 (15-29) and G5 (<15, or treated by dialysis/renal transplant). Ethical approval was obtained from Kenyatta National Hospital-University of Nairobi Ethics and Research Committee (KNH-UoN ERC), approval number P510/09/2017. We estimated prevalence of CKD and used logistic regression to determine factors independently associated with CKD diagnosis.

**Results:**

We interviewed 306 inpatients; median age 40.0 years (IQR 24.0), 162 (52.9%) were male, 155 (50.7%) rural residents. CKD prevalence was 118 patients (38.6%, 95% CI 33.3-44.1); median age 42.5 years (IQR 28.0), 74 (62.7%) were male, 64 (54.2%) rural residents. Respondents with CKD were older than those without (difference 4.4 years, 95% CI 3.7-8.4 years, P = 0.032). Fifty-six (47.5%) of the patients had either stage G1 or G2, 17 (14.4%) had end-stage renal disease; 64 (54.2%) had haemoglobin below 10g/dl while 33 (28.0%) had sodium levels below 135 mmol/l. ). History of unexplained anaemia (aOR 1.80, 95% CI 1.02-3.19), proteinuria (aOR 5.16, 95% CI 2.09-12.74), hematuria (aOR 7.68, 95% CI 2.37-24.86); hypertension (aOR 2.71, 95% CI 1.53-4.80) and herbal medications use (aOR 1.97, 95% CI 1.07-3.64) were independently associated with CKD.

**Conclusion:**

Burden of CKD was high among this inpatient population. Haematuria and proteinuria can aid CKD diagnosis. Public awareness on health hazards of herbal medication use is necessary.

## Introduction

Chronic kidney disease (CKD) is defined as decreased glomerular filtration rate (GFR) of less than 60 mL/min per 1.73 m^2^ for more than 3 months, with or without kidney damage; or functional or structural abnormalities of the kidneys with or without decreased GFR [1, 2]. It is classified into five stages, with the severest form being end-stage renal disease which requires renal replacement therapy either in the form of dialysis or renal transplantation [2]. The main risk factors for CKD globally are diabetes mellitus, hypertension and glomerulonephritis; other associations include genetics, family history, gender, increasing age smoking and nephrotoxins [3-5]. The global burden of chronic kidney disease is estimated to be 11 to 13% [6]. The prevalence of renal disease in Africa is not known, though estimates point to a substantial burden especially in the middle-aged [7]. Prevalence of CKD in specific health conditions has previously been estimated in Kenya, including in HIV, rheumatoid arthritis, heart failure and type 2 diabetes [8-12]. However, little is known on overall prevalence of CKD in the Kenyan population; such data would be invaluable in informing public health investment in treatment facilities and prevention. The gold standard for determining CKD burden is population surveys; however these are difficult due to time and financial constraints. Prevalence of CKD in medical inpatients at tertiary/referral health facilities has been used in several studies in different countries and settings to estimate the overall disease burden and its complications. These include Uganda, Botswana and the United States [13-15]. Our study aimed to estimate the prevalence and identify factors associated with CKD among medical inpatients at the largest tertiary hospital in Kenya.

## Methods

**Study design:** this was a cross-sectional study among medical inpatients at a tertiary health facility.

**Study setting:** the study was conducted at the Kenyatta National Hospital, a national referral facility based in an urban setting. The health facility has eight internal medicine wards, with a combined medical inpatient capacity of 640 patients.

**Participants:** the study participants were all patients, above 18 years, admitted in any of the medical wards at the Kenyatta National Hospital, in March 2018 who met the eligibility criteria.

### Eligibility criteria

***Inclusion criteria:*** all patients admitted in the medical wards, above the age of 18 years, who gave consent, were eligible for inclusion.

***Exclusion criteria:*** the following patients were excluded from the study: 1) Patients with cognitive dysfunction; 2) Absence of requisite laboratory or imaging results: creatinine (for renal dysfunction determination), markers of CKD for those with abnormal creatinine (calcium, phosphate, proteinuria or typical findings on renal ultrasound), or CKD complications (full blood count); 3) Patients with extreme fatigue or weakness for data tool administration, especially for patients with marked fluid overload, dyspnoea or voice impairment.

### CKD diagnosis

A diagnosis of CKD was defined as presence of decreased glomerular filtration rate (GFR) below 60 mL/min per 1.73m^2^, or GFR above 60 mL/min per 1.73 m^2^ but with markers of renal damage, as determined by the Chronic Kidney Disease Epidemiology collaboration (CKD-Epi) equation [16] and at least one of the following: 1) Contracted kidneys, hypo-echoic kidneys or loss of corticomedullary differentiation on renal imaging; 2) Serum phosphate levels above 1.4mmol/l; 3) Serum calcium level below 2.2mmol/l; 4) History of kidney transplantation; 5) History of documented kidney dysfunction for more than 3 months; 6) Anaemia of chronic disease (normochromic, normocytic, hypo-proliferative picture), with haemoglobin level of <10/dl [17].

### Sample size assumptions and calculations

The sample size was calculated using the Cochrane formula [18], with the following assumptions: Z statistic of 1.96 for 95% confidence level, expected prevalence of CKD of 27% in a study done in similar settings and a precision of 0.05. The minimum sample size required was 303.

### Sampling methods

A pre-visit was conducted before the commencement of the study to ascertain the patients on admission per ward at the time of study. The number of participants for sampling from each ward was allocated equally since the wards have the same capacity; this was determined by dividing the total sample size with the number of medical wards at the study site.

*The number to be sampled from each ward=Total sample size (303) divided by number of medical inpatient wards (8) = 38 patients.*

Systematic random sampling with replacement was used to select the 38 patients from each ward. The sampling frame was the ward inpatient register, which records all the patients on admission at any time and the numbers to track their inpatient records. The sampling interval was determined as follows:

*Sampling interval (K) = No. of patients admitted in that particular ward at beginning of sampling divided by number to be sampled from each ward.*

A number between the first and the K^th^ patient was selected as the first participant to be sampled and thereafter sampled very K^th^ patient. This was repeated till the required sample size from that ward was achieved; the procedure was then repeated in all the eight wards. Sampling with replacement was carried out by obtaining consent from the next sequential inpatient meeting the case definition in case the one selected opted out of the interview.

### Data collection

Data on socio-demographic information, behavioural, family and medical history were collected using structured paper questionnaires administered through face to face interviewing of the study participants. The questionnaire was pretested on 30 patients selected from the general medical outpatient clinic. Data on laboratory and imaging investigations were collected from the inpatient medical files.

### Data management and statistical analysis

The data from the paper questionnaires were entered into a computer, cleaned, coded and loaded it into statistical software for analysis. We derived means for continuous variables and proportions to describe the socio-demographic characteristics of the study participants as well as the prevalence of CKD. After calculating GFR by the CKD-Epi equation to diagnose CKD, the patients were classified into five stages using GFR in ml/min/1.73m^2^ G1 (≥90), G2 (60-89), G3a (45-59), G3b (30-44), G4 (15-29) and G5 (<15, or treated by dialysis/renal transplant). Using selected variables as exposures and CKD case status as the outcome, we carried out bivariate analysis, and subsequently included factors with P-value below 0.15 into an unconditional logistic regression model, employing the forward selection approach and level of significance of 0.05. This was achieved by selecting the independent variables with P-value below 0.15 at bivariate analysis, ranked them from the smallest to the biggest, then run them in the model sequentially as per the rank. Factors that became insignificant at 0.05 level when first introduced into the model were removed; the model was run several times till the best fit was achieved.

### Ethical considerations

We obtained written informed consent form every study participant, removed personal identifiers and maintained strict confidentiality of the collected data using lockable cabinets for paper questionnaires and password-protected computer for the electronic database. The study was approved by the Kenyatta National Hospital-University of Nairobi Ethics and Research Committee (KNH-UoN ERC), number P510/09/2017.

## Results

### Socio-demographic characteristics

We interviewed 306 medical inpatients, originating from 33 of the 47 Kenyan counties. The median age of the respondents was 40.0 years (IQR 24.0 years), 162 (52.9%) were male, 155 (50.7%) were rural residents, 157 (51.3%) had at least completed secondary education, 69 (22.6) % were on full-time employment and 182 (59.5%) were married (Table 1).

**Table 1:** Socio-demographic characteristics of the selected Medical inpatients, Kenyatta National Hospital, Kenya, 2018 (n=306)

Variable	Categories	Frequency (%)
Sex	Female	144 (47.1)
	Male	162 (52.9)
Age (years)	Less than 40	141 (46.1)
	40 and above	165 (53.9)
Residence	Urban	151 (49.3)
	Rural	155 (50.7)
Education level	No formal education	19 (6.2)
	Primary incomplete	40 (13.1)
	Primary complete	55 (18.0)
	Secondary incomplete	35 (11.4)
	Secondary complete	91 (29.7)
	College and above	66 (21.6)
Marital status	Single	66 (21.6)
	Married	182 (59.5)
	Separated	20 (6.5)
	Divorced	9 (2.9)
	Widowed	29 (9.5)
Occupation	Unemployed	86 (28.1)
	Homemaker	33 (10.8)
	Part-time employment	96 (31.4)
	Fulltime employment	69 (22.6)
	Retired	22 (7.2)

### CKD prevalence and electrolyte abnormalities

**Figure 1:**
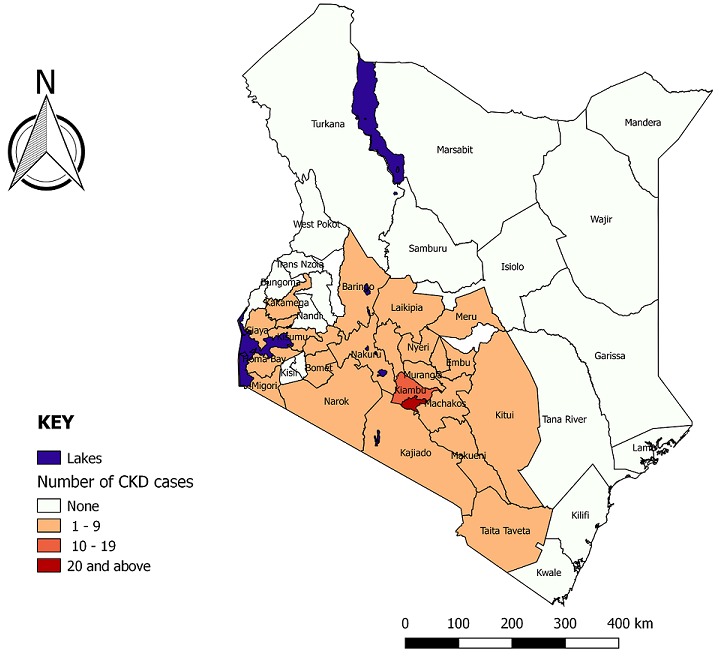
Chronic kidney disease cases by counties of origin, Kenyatta National Hospital, 2018

Of the 306 respondents, 118 had CKD, translating to a prevalence of 38.6% (95% CI 33.3, 44.1). The median age of the respondents with CKD was 42.5 years (IQR 28 years). Respondents with CKD were older than those without (age difference 4.4 years, 95% CI 3.7-8.4 years, P = 0.032; 74 (62.7%) were male while 64 (54.2%) were rural residents. Patients with CKD originated from 26 of the 47 Kenyan Counties, spanning the coastal, central, rift valley and Western parts of the country (Figure 1). Classified by GFR stage, 20 (16.9%) of the patients had stage G1, 36 (30.5%) had stage G2, 23 (19.5%) had stage G3a, 11 (9.3%) had stage G3b, 11 (9.3%) had stage G4 and 17 (14.4%) had end-stage renal disease. Advanced CKD (stage G4 and G5) was present in 28 (23.7%) of the CKD patients. Among the CKD patients, 73 (61.9%) had hemoglobin levels below 10g/dl and 33 (28.0%) had hyponatremia (serum sodium <135mmol/l) (Table 2).

**Table 2:** Prevalence of hematologic and electrolyte abnormalities among the chronic kidney disease patients, Kenyatta National Hospital, Kenya, 2018

Derangement	Frequency (%)		
	Overall (n=118)	Male (n=74)	Female (n=44)
Anaemia Hb <10g/dl	73 (61.9)	43 (58.1)	30 (68.2)
Serum sodium below 135 mmol/l	33 (28.0)	21 (28.4)	12 (27.3)
Serum Potassium above 5 mmol/l	22 (18.6)	13 (17.6)	9 (20.5)
Serum phosphate >1.4 mmol/l	11 (9.3)	7 (9.5)	4 (9.1)
Serum calcium below 2.2 mmol/l	6 (5.1)	5 (6.8)	1 (2.3)

Hb: haemoglobin

### Bivariate analysis

Males had higher odds of being diagnosed with CKD compared to females (OR 1.91, 95% CI; 1.19, 3.06). Participants with previous history of diabetes (OR 1.89, 95%; 95% CI; 1.12, 3.18) and hypertension (OR 3.57, 95% CI; 2.16, 5.91) had increased odds of CKD diagnosis compared with those without. Current or past smokers had higher odds of CKD diagnosis compared to non-smokers (OR 1.76, 95% CI; 1.08-2.88), as were those with history of alcohol use (OR 2.10, 95% CI; 1.32, 3.36). Participants who had episodes of the following symptoms at any time before their diagnosis had higher odds of CKD diagnosis: haematuria (OR 13.00, 95% 4.29, 52.35) recurrent urinary tract infections (OR 2.33, 95% CI; 1.37, 3.95), proteinuria (OR 8.73, 95% CI 4.02, 18.97), kidney stones (OR 8.61, 95% CI; 1.78, 81.72) or cardiovascular disease (OR 1.76, 95% CI; 1.09, 2.86) (Table 3).

**Table 3:** Factors associated with being diagnosed with chronic kidney disease case, Kenyatta National Hospital, Kenya, 2018 (n=306)

Variable	Response	CKD n (%)	No CKD n (%)	OR (95% CI)	aOR (95% CI)
Age ≥40 years	Yes	69 (58.5)	96 (51.1)	1.35 (0.85, 2.15)	
	No	49 (41.5)	92 (48.9)	Ref	
Sex	Male	74 (62.7)	88(46.8)	1.91 (1.19, 3.06)	2.63 (1.49, 4.63)
	Female	44(37.3)	100(53.2)	Ref	
Any chronic disease	Yes	99(83.9)	134(71.3)	2.10 (1.17, 3.76)	1.28 (0.64, 2.57)
	No	19(16.1)	54(28.7)	Ref	
Cancer	Yes	15(12.7)	30(16.0)	0.77 (0.39, 1.50)	
	No	103(87.3)	158(84.0)	Ref	
Circulation disorders	Yes	39(33.1)	39(20.7)	1.89 (1.12, 3.18)	
	No	79(66.9)	149(79.3)	Ref	
Type 2 diabetes	Yes	31(26.3)	29(15.4)	1.95 (1.11, 3.45)	1.29 (0.66-2.52)
	No	87(73.7)	159(84.6)	Ref	
Hypertension	Yes	57(48.3)	39(20.7)	3.57 (2.16, 5.91)	2.71 (1.53, 4.80)
	No	61(51.7)	149(79.3)	Ref	
HIV infection	Yes	20(17.0)	40(21.3)	0.76 (0.42, 1.37)	
	No	98(83.0)	148(78.7)	Ref	
Nephrotoxic drug use	Yes	84(71.2)	136(72.3)	0.94 (0.57, 1.57)	
	No	34(28.8)	52(27.7)	Ref	
NSAIDS	Yes	70(59.3)	110(58.5)	1.03 (0.65, 1.65)	
	No	48(40.7)	78(41.5)	Ref	
Tobacco use	Yes	46(39.0)	50(26.6)	1.76 (1.08, 2.88)	0.89 (0.47, 1.69)
	No	72(61.0)	138(73.4)	Ref	
Relative with renal disease	Yes	17(14.4)	14(7.5)	2.09 (0.99, 4.42)	0.90 (0.33-2.46)
	No	101(85.6)	174(92.5)	Ref	
Alcohol use	Yes	70(59.3)	77(41.0)	2.10 (1.32, 3.36)	1.37 (0.81, 2.31)
	No	48(40.7)	111(59.0)	Ref	
CVD	Yes	49(41.5)	54(28.7)	1.76 (1.09, 2.86)	1.41 (0.76-2.63)
	No	69(58.5)	134(71.3)	Ref	
Residence	Urban	97(51.6)	54(45.8)	1.26 (0.80, 2.00)	
	Rural	91(48.4)	64(54.2)	Ref	
Recurrent UTI^a^	Yes	41(34.8)	35(18.6)	2.33 (1.37, 3.95)	1.23 (0.65, 2.32)
	No	77(65.2)	153(81.4)	Ref	
Hematuria history^a^	Yes	26(22.0)	4(2.1)	13.00 (4.29, 52.35)	7.68 (2.37, 24.86)
	No	92(78.0)	184(97.9)	Ref	
Herbal medications use^a^	Yes	34(28.8)	37(19.7)	1.65 (0.97, 2.83)	1.97 (1.07, 3.64)
	No	84(71.2)	151(80.3)	Ref	
History of proteinuria^a^	Yes	36(30.5)	9(4.8)	8.73 (4.02, 18.97)	5.16 (2.09, 12.74)
	No	82(69.5)	179(95.2)	Ref	
History of Kidney stones^a^	Yes	10(8.5)	2(1.1)	8.61 (1.78, 81.72)	0.67 (0.09, 5.29)
	No	108(91.5)	186(98.9)	Ref	
History of anaemia^a^	Yes	57(48.3)	58(30.9)	2.09 (1.30,3.37)	1.80 (1.02, 3.19)
	No	61(51.7)	130(69.1)	Ref	

a These symptoms were prior to diagnosis of chronic kidney disease or current admission for the incident cases

b: deep venous thrombosis or peripheral vascular disease, OR: odds ratio, aOR: adjusted odds ratio, NSAIDS: non-steroidal anti-inflammatory drugs, CVD: cardiovascular disease, UTI: urinary tract infection, Ref: reference group, NS: non-significant

### Multivariable analysis

Male sex (aOR 2.63, 95% CI; 1.49, 4.63), previous history of haematuria (aOR 7.68, 95% CI; 2.37, 24.86), anaemia (aOR 1.80, 95% CI; 1.02, 3.19) proteinuria (aOR 5.16, 95% CI; 2.09, 12.74), hypertension (aOR 2.71, 95% CI; 1.53, 4.80) and herbal medication use (aOR 1.97, 95% CI; 1.07,3.64) were independently associated with being diagnosed with CKD (Table 3).

## Discussion

This health facility-based study revealed a high burden of CKD in the inpatient population, with a prevalence of approximately 4 out of 10 inpatients. Anaemia and low serum sodium were the most common abnormalities among the CKD patients. On staging, approximately half of the CKD cases in our study had mild disease (stage G1 and G2) while a quarter had advanced disease. Male sex, previous history of haematuria, proteinuria, anaemia, hypertension and use of herbal medications as factors associated with CKD in this inpatient population.

Similar studies done in different countries reported a lower prevalence of CKD compared to our study; 15.3% in Uganda, 16.3% in Botswana and 22.3% in the US [13-15]. This could suggest a higher disease burden in our settings or differences in the approach of these studies. The Ugandan study, for instance, performed ultrasonography in all the included patients, hence likely increasing specificity and restriction of their case definition; in our study, only 10% had the imaging done, with features suggestive of CKD present in 5.1%. The Botswana study had a similar case ascertainment to our study, but still had lower burden of disease. The US study had a mixed-race population, but the prevalence of CKD among African Americans, was 28.1%, still lower than our study, suggesting different disease burdens, possible different environmental factors or differences in case ascertainment process.

Other studies done in Kenya on prevalence of CKD in specific conditions have found prevalence ranging from 17.6% among HIV patients on HAART to 54.5% among ambulatory type 2 diabetic patients [9, 11]. Diabetes is a major risk factor for CKD, therefore could explain the prevalence higher than our study. The CKD cases originated from 27 out of 47 Kenyan Counties, all along the Northern corridor. This is likely due to the fact that majority of the Kenyan population live along this particular corridor; the ease of accessibility also may have made it easier for people from these regions to seek specialized care at the tertiary health facility. Majority of the CKD cases were male; while CKD generally is more prevalent in women, severe forms are higher in men [19]. One suggested mechanism is role of testosterone and protective function of oestrogen in women [20]. Majority of the CKD cases were middle-aged, and were significantly older than their non-CKD counterparts. Older age is also a recognized risk factor for CKD [5, 21]. One explanation is that renal function generally decreases with age; hence older individuals are more prone to CKD after renal injury. In our study, most of the cases were rural residents. A systematic review and meta-analysis of studies on CKD in Sub-Saharan Africa did not find any difference in prevalence between rural and urban populations [22]. The Kenya STEPS survey 2015 did not find significant differences between rural and urban prevalence of diabetes and hypertension and since majority of the population are rural residents, this could explain higher disease burden in this demographic [23]. Another possible explanation could be affordability; richer, urban dwellers are likely to prefer private hospitals rather than the public tertiary facility.

Over half of the CKD patients had anaemia with haemoglobin below 10g/dl. A study done in Kenyan study found a comparable prevalence of anaemia in CKD patients, at 67% [24]. Different mechanisms cause anaemia in CKD, including erythropoietin deficiency, uremic inhibition of erythropoiesis, shortened red cell lifespan and hepcidin excess [25]. Other electrolyte imbalances in our study included low sodium, elevated potassium levels and abnormal bone metabolism (low calcium or elevated serum phosphate levels). These have been observed in other studies, with hyperkalaemia attributed to reduced kidney function, hyponatremia a consequence of fluid retention and loss of renal regulation of calcium and phosphate homeostasis [26]. High prevalence of these abnormalities in our study may point to low early detection and management in clinical settings; failure to properly manage these complications can worsen CKD and quality of life [27].

On staging, approximately half of the CKD cases in our study had mild disease, a quarter had stage G4 or G5 (advanced disease) while one in seven patients had end-stage disease (ESRD). Two previous Kenyan studies found prevalence of ESRD of 32% and 11.5% [24, 28]. These studies however were focussing on a population of already diagnosed CKD patients primarily, in renal clinics. Another Kenyan study focusing on a population of type 2 diabetes without prior diagnosis of CKD reported a prevalence of ESRD of 0.5% while the Ugandan study reported 4.6% [9, 15]. Our study was comparable in approach to the Ugandan study and therefore may indicate either higher prevalence in our settings or differences in how case detection was made. Since this is the stage that requires renal replacement therapy, our study provides pilot data for planning population-based surveys for mapping of regional and national burden as well as need for investment in advanced renal treatment infrastructure.

Our study found male sex, having been diagnosed with haematuria, proteinuria, anaemia or hypertension at some time in the past (preceding CKD diagnosis) and use of herbal medications as factors associated with CKD in this inpatient population. In comparison, a study in India reported association of CKD with anaemia [29]. Another study in Asia found association with male sex [30]. Association with urinary proteins has also been reported [31]. Use of herbal medications has been previously associated with acute kidney injury (AKI) which is a recognized precursor of CKD [32]. Other suggested mechanisms of herbal medications role in CKD include direct nephrotoxicity augmented by underlying predisposing conditions such as dehydration; contamination, or adulteration of remedies; inappropriate use or preparation or interactions with other medications [33]. Haematuria has been identified as frequent manifestation of glomerular disease, a forerunner of CKD and may have a role in diagnosing early renal damage [34].

### Study limitations

Some signs (blood/proteins in urine) could have occurred after CKD process. To ensure we captured only symptoms experienced prior to diagnosis, we corroborated the information from the interviews with the medical history captured in the inpatient records. Interpretation of our results does not intend to infer causality; rather the association between the two early symptoms and CKD may have a role in early diagnosis. There was also possibility of misclassifying AKI as CKD; however, our study only considered those with reduced GFR and presence of at least one other feature to help differentiate it from AKI, we believe this increased the specificity of the diagnostic criteria.

## Conclusion

There was high burden of CKD in medical inpatients at this Kenyan tertiary facility. Anaemia and electrolyte abnormalities were common among patients with CKD. Male sex, previous documented history of unexplained anaemia, hematuria or proteinuria and herbal medications use were identified as associated factors.

### Recommendations

Early diagnostic work-up for CKD with urinalysis should be advised for primary care settings as possible early markers of CKD. Protein or blood in urine on urinalysis, especially in males, should serve as referral criteria for more extensive workup. Public education on dangers of herbal medications should be carried out at the national level, emphasizing their association with CKD diagnosis. Our study findings could serve as pilot data for planning of population-based surveys, both in a rural and urban setting, to better characterize the burden of CKD in Kenya.

### What is known about this topic

- Prevalence of CKD in various medical conditions, including hypertension, diabetes type 2 and HIV;
- CKD risk factor prevalence in the Kenyan general population is known.

### What this study adds

- An estimate of the population CKD burden, in the absence of population-based survey data;
- Associations amenable to public health intervention.

## Competing interests

The authors declare no competing interests.
